# Translation and psychometric properties of the MISSCARE survey-Persian version

**DOI:** 10.1186/s12912-021-00787-w

**Published:** 2022-01-04

**Authors:** Zeinab Hosseini, Leila Raisi, Amirhossein Maghari, Mansoureh Karimollahi

**Affiliations:** 1 School of Nursing and Midwifery, Ardabil University of Medical Sciences, Ardabil, Iran; 2 Department of Midwifery, School of Nursing and Midwifery, Ardabil University of Medical Science, Ardabil, Iran; 3 Department of Family Health, Social Determinants of Health Research Center (SDHRC), Ardabil University of Medical Science, Ardabil, Iran; 4 Atherosclerosis Research Center, Baqiyatallah University of Medical Sciences, Tehran, Iran; 5 Department of Nursing, Faculty of Nursing and Midwifery, Ardabil University of Medical Sciences, Ardabil, Iran

**Keywords:** Nursing, Patients, Missed care, Translation, Psychometrics

## Abstract

**Background:**

Providing safe and high-quality nursing care is an essential task of nursing. Nurses may be unable to provide patients with all of the necessary care for numerous reasons, such as an increase in the number of patients and a low number of nursing staff. Moreover, they may have to omit, postpone, or incompletely perform a series of care, referred to as missed nursing care. The purpose of this study was to translate and conduct psychometric testing of the MISSCARE Survey.

**Method:**

In this study, we accurately translated the MISSCARE Survey. Its acceptability, construct validity, and internal consistency were analyzed. This cross-sectional study was conducted in the summer of 2020 in educational hospitals in Ardabil, Iran. The participants were 300 nurses who worked in educational hospitals and were randomly selected.

**Results:**

Participants in this study included 300 nurses from five units, including general medicine (13.3%), COVID-19 (45.0%), surgery (18.7%), critical care unit (6.3%), and intensive care unit (16.7%), who worked various shifts, of whom 84.7% were female. The total content validity in Part A was 0.944, and that in Part B was 0.969. Part A was divided into three domains (necessary care, secondary care, and supportive care), and Part B was divided into five domains (communication, labor resources, material resources, responsibility, and unpredictable situations). In both parts, the chi-square index was < 3, and the RMSEA index was < 0.08. The internal consistency measured by Cronbach’s alpha was 0.933 for Part A and 0.910 for Part B for the Persian version of the MISSCARE Survey.

**Conclusion:**

Based on the outcomes of this research, it can be concluded that the Persian version of the MISSCARE Survey is valid for use in Iranian hospitals and can be used to identify missed care and the reasons behind it. Nursing managers can also use it to improve the situation and provide the highest-quality care.

## Background

Nursing care is a skillful, safe, high-quality, ethical, and shared-care process designed and planned based on the best clinical evidence supporting the patient’s health, symptom relief, or a quiet death [1]. Ensuring patient health and the quality of nursing care are fundamental challenges for nursing managers [2]. Many studies have shown a relationship between the performance of nursing staff and the quality of patients’ health [3, 4]. On the other hand, the hospital work environment involves rapid and unpredictable events that can lead to disruptions and mistakes in nursing care because nurses constantly move from one activity to another and manage multiple sources of care [5]. Sometimes, nurses cannot address all care requests or may not complete all aspects of care for various reasons. In such situations, nurses may reduce, delay, or eliminate care. These cases are more pronounced, particularly in the current circumstances and the COVID-19 epidemic era, and with the increase in the volume of patients and the increase in the workload, less care may be provided.

Missed nursing care refers to any aspect of care that is partially or completely eliminated or delayed [6]. Kalisch first identified the phenomenon of missed nursing care in a qualitative study [2]. Nurses are trained in standards of care in basic nursing education programs, and this learning is reinforced through on-the-job training and assessment systems. However, nurses reported that some aspects of nursing care were missed regularly. The findings of this study point to two critical issues in nursing: the patient care provided is less than what nurses have learned and less than what patients need to recover [7]. Further, when nurses are unable to fulfill their responsibilities to meet the patient’s needs, they feel dissatisfied with themselves and their work.

According to Bowles and Candel, nurses who have negative perceptions of their work experience may leave their jobs at the earliest opportunity, which exacerbates the lack of organizational resources and the use of resources for employment and acquaintance for replacement [8]. Therefore, it is necessary to use a tool that can examine the extent and nature of this phenomenon and make it possible for employers to study the percentage of eliminated care in addition to the reasons for such negligence. For this purpose, the MISSCARE Survey was developed to measure missed nursing care and the reasons behind it. The tool was developed in the United States and has two parts: Part A, which contains 24 elements related to primary nursing care categorized as “educating the patient about disease, procedures, and diagnostic studies,” and Part B, which contains 17 reasons for missed care categorized as an “inappropriate ratio of patients to nurses” [2]. The MISSCARE Survey has been evaluated in many countries, such as Brazil, Slovakia, Sweden, and Turkey, and was deemed acceptable in all of those countries [9–13]. Previous studies have shown that ambulation, turning, delayed or missed feedings, and emotional support were missed more frequently than other types of care [9–13]. According to the literature, although it is essential to have a tool for measuring missed nursing care, a study to validate the MISSECARE Survey has not been conducted in Iran. Thus, the purpose of this study was to translate and conduct psychometric testing of the MISSCARE Survey.

## Method

### Design

The design of the study was a cross-sectional, descriptive survey.

### Characteristics of the MISSCARE survey

Kalisch et al. developed the MISSCARE Survey in 2009 in the United States. It consists of three parts: the first part includes demographic information; the second part (Part A) includes 24 items, a list of nursing care activities, which are answered on a 5-point Likert scale ranging from “always missed” to “never missed.” The third part (Part B) includes 17 items regarding the reasons for missed care, which are answered on a 4-point Likert scale ranging from “significant reason” to “NOT a reason for missed nursing care.”

### Procedure

#### Translation of the MISSCARE survey

First, we obtained official approval from the author to use the MISSCARE Survey. The translation of the MISSCARE Survey was accomplished according to World Health Organization (WHO) guidelines, including translation, the use of an expert panel, back-translation, cognitive interviewing, and a finalization process.

In the first step, we translated the survey with two independent expert translators; then, in a group that included two nursing faculty members and the authors, we combined the two versions of the translation into a single version. Next, we translated that version back into English with a native English translator who was fluent in Persian. We then compared the translated English and original versions with two other independent translators. A panel of five experienced nurses evaluated the relevance and applicability of the translated versions and created the final version of the MISSCARE Survey- Persian.

Translating an instrument into another language and culturally adapting it to a new context is time-consuming and requires great effort from both the researchers and the healthcare professionals. Therefore, we sought help from some other researchers who were experts in both languages and had the same experience.

The MISSCARE Survey- Persian version was given to ten professionals and nursing experts for evaluation of its content and face validity. We used the content validity ratio (CVR) and content validity index (CVI) to evaluate content validity. To determine the CVR, we asked experts to read each item and choose one of the following options: “Essential and useful,” “Useful but not essential,” or “Not essential.” The items selected as “Essential and useful” were used to calculate and compare the survey to the Lawshe table [14]. In this study, we had ten experts. Thus, the minimum CVR for each item was 0.62. The CVR for all items in Parts A and B of the survey was greater than 0.62.

To determine the CVI, we asked experts to select “Fully related,” “Related,” “Somewhat related,” or “Irrelevant” for each item. The items selected as “Fully related” and “Related” were used in the study. The minimum necessary score for each item was 0.79 [15]. In this study, the CVI for Part A was 0.944, and that for Part B was 0.969, much higher than the minimum necessary score.

Face validity was calculated after determining impact score of each question. Items with an impact score of more than 1.5 are considered valid [16]. The face validity for all items in both parts of the survey was more than 1.5. Thus, the survey is considered valid.

#### Participants

Using the consensus sampling method, we chose 300 nurses from five units (general medicine, surgery, intensive care unit (ICU), cardiac care unit (CCU), and COVID-19) in four educational hospitals. Participants had at least a bachelor’s degree in nursing and at least six months of experience in nursing with no mental problems, and the response rate was approximately 60%.

### Data collection

The MISSCARE Survey- Persian version was used for data collection, which began in February 2019. However, we had to halt collection in late February because of the COVID-19 pandemic. Collection was resumed in June 2020 and completed in September 2020.

### Data analysis

We used Excel 2013 to evaluate content and face validity. Construct validity was assessed using confirmatory factor analysis (CFA) with maximum likelihood estimation using AMOS v. 21. Cronbach’s alpha and McDonald’s omega coefficient were used to assess the reliability of the survey. IBM SPSS version 21 and the “omega” function of the “psych” package in R (4.0.2) software were used for statistical analysis and *p* < 0.05 was considered statistically significant.

## Results

The participants were 300 nurses from five units: COVID-19 (45%), surgery (18.7%), ICU (16.7%), CCU (6.3%), and general medicine (13.3%). Most of the participants (84.7%) were women; most (49.3%) were 25–34 years old, while 39.7% were 35–44 years old. The majority of the participants (97.3%) had at least a bachelor’s degree in nursing, and 99.3% worked as staff nurses. In this study, 44.4% of the participants had more than five years of work experience, while 55.6% had less than five years of work experience.

### Confirmatory factor analysis

We divided the survey into different domains. Part A included necessary care, secondary care, and supportive care. Part B included communication, material resources, labor resources, responsibility, and unpredictable situations.

**Fig. 1:**
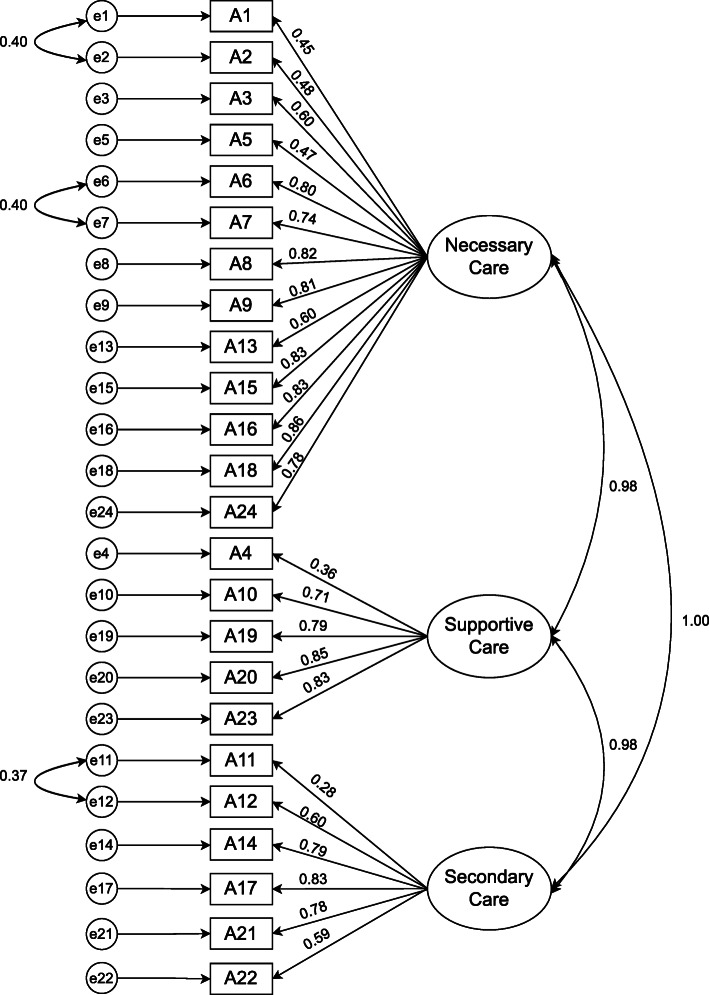
Coefficients Standardized (Part A)

**Fig. 2:**
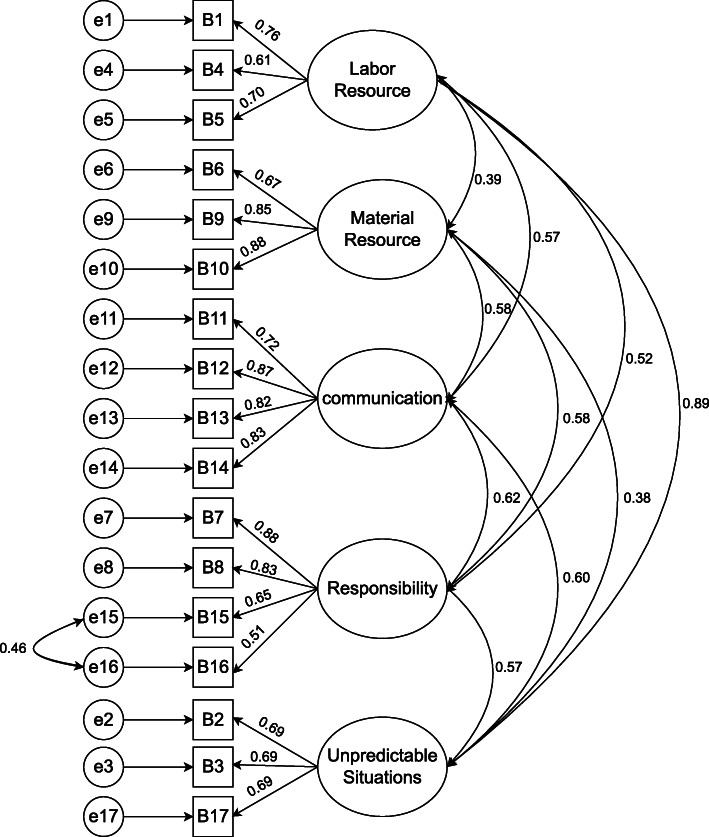
Coefficients Standardized (Part B)

The result of construct validity testing via CFA in both parts A and B of the survey showed that the scaled chi-square with a degree of freedom was less than 3 and the RMSEA was less than 0.08 (Table 1). Therefore, the resulting model was confirmed (Figs. 1 and 2).

**Table 1:** Summary of the results of confirmatory factor analysis

	Chi-Square	df	Chi-Sq./df	*P*-value	GFI	GFI	CFI	NFI	RMSEA
Missed care	593.57	245	2.419	0.364	0.934	0.908	0.900	0.906	0.069
Reason for Missed care	285.998	108	2.648	0.260	0.959	0.907	0.934	0.900	0.074

Tables 2 and 3 present the analysis of the two parts of the survey. The results showed that the relationship between all items and their structure or factors was significant, and all items had the necessary structural validity. In addition, all structures showed a significant correlation, which indicates a logical relationship between the structures in the building of the survey (Parts A and B). Finally, the construct validity of the survey was confirmed for both Parts A and B.

**Table 2:** Summary of the results of items and connection between factors and items (Part A)

Factors	Item	Standardized B	Coefficients B	SD Error	*P* value
Necessary care	Moving patient three times a day or as prescribed	0.455	1.000		
	Changing the patient’s position every 2 h	0.482	0.876	0.133	0.001
	Serving a warm dish to the patient	0.602	1.257	0.188	0.001
	Giving medications 30 min before or after a schedule	0.470	0.913	0.162	0.001
	Taking vital signs as prescribed	0.805	1.500	0.229	0.001
	Control of intake and output	0.745	1.352	0.212	0.001
	Complete recording of all necessary data	0.827	1.458	0.238	0.001
	Educating the patient about disease, procedures, and diagnostic studies	0.815	1.730	0.279	0.001
	Washing hands	0.604	1.297	0.229	0.001
	Patient’s blood sugar monitoring as prescribed	0.837	1.789	0.295	0.001
	Evaluating the patient in each shift	0.839	1.795	0.282	0.001
	Evaluating peripheral and central venous catheter based on hospital protocols	0.867	1.791	0.293	0.001
	Skin/ wound care	0.783	1.893	0.300	0.001
Supportive care	Setting table for patients who eat by themselves	0.368	1.000		0.001
	Emotional support of patient and/or family	0.718	2.119	0.507	0.001
	Responding to the patient alarm within 5 min	0.712	2.344	0.566	0.001
	Administrating PRN medication within 15 min	0.852	2.505	0.638	0.001
	Assisting the patients with toileting within 5 min after a request	0.830	2.574	0.623	0.001
Secondary care	Emotional support of patient and/or family	0.282	1.000		
	Mouth care	0.605	2.194	0.493	0.001
	Discharge planning and the patient educating	0.794	3.534	0.964	0.001
	Focused reevaluation of the patient considering the patient’s condition	0.836	3.456	0.971	0.001
	Evaluation of drug effects	0.783	3.552	0.969	0.001
	Attending interdisciplinary caring conferences	0.594	2.456	0.692	0.001
Connection and correlation		Standardized B	**C**oefficients B	SD Error	P
Necessary care	Supportive care	0.980	0.112	0.038	0.003
Supportive care	Secondary care	0.976	0.059	0.024	0.015
Necessary care	Secondary care	0.998	0.084	0.029	0.004
measurement error of A11	measurement error of A12	0.377	0.161	0.035	0.001
measurement error of A2	measurement error of A1	0.397	0.198	0.041	0.001
measurement error of A7	measurement error of A6	0.399	0.085	0.017	0.001

**Table 3:** Summary of the results of items and connection between factors and items (Part B)

Factor	Item	Standardized B	Coefficients B	SD Error	P
Labor resource	Low nurse staffing	0.763	0.863	0.079	0.001
	The low number of assistant and office staff (for example assistant nurse, technician, secretary, etc.)	0.618	0.866	0.093	0.001
	Inappropriate rate of patient to nurse	0.705	1.000		
Material resource	Unavailability of drugs when needed	0.671	0.785	0.062	0.001
	Unavailability of needed devices	0.859	0.954	0.055	0.001
	Dysfunctionality of devices as needed	0.885	1.000		
Communication	Not supporting by team members	0.726	0.840	0.061	0.001
	Tension or miscommunication with other wards	0.870	0.983	0.056	0.001
	Tension or miscommunication in the nursing team	0.824	1.067	0.065	0.001
	Tension or miscommunication with medical staff	0.830	1.000		
Responsibility	Inappropriate transfer from a previous shift or other wards	0.881	1.597	0.178	0.001
	Not giving required cares by other wards (for example the Physiotherapist has not moved the patient)	0.831	1.505	0.171	0.001
	Not informing assistant nurse about missed cares	0.658	1.197	0.115	0.001
	Leaving the ward or unavailability of nurse	0.510	1.000		
Unpredictable situations	Emergency conditions (for example aggravation of the patient’s condition)	0.694	1.020	0.101	0.001
	The unexpected increase in the number of patients or workload in the unit	0.691	0.963	0.095	0.001
	The high number of hospitalizing and discharging	0.691	1.000		
Connection and correlation					
Labor resource	Material resource	0.390	0.204	0.041	0.001
Labor resource	Communication	0.575	0.296	0.045	0.001
Labor resource	Responsibility	0.515	0.161	0.031	0.001
Labor resource	Unpredictable situations	0.886	0.339	0.045	0.001
Material resource	Communication	0.576	0.409	0.055	0.001
Material resource	Responsibility	0.680	0.293	0.045	0.001
Material resource	Unpredictable situations	0.381	0.201	0.042	0.001
Communication	Responsibility	0.622	0.265	0.043	0.001
Communication	Unpredictable situations	0.600	0.312	0.046	0.001
Responsibility	Unpredictable situations	0.572	0.181	0.033	0.001
measurement error of B16	measurement error of B16	0.456	0.272	0.042	0.001

### Internal consistency

Cronbach’s alpha and McDonald’s omega coefficients were used to determine the reliability of the survey. The results showed that all three factors of Part A and all five factors of Part B had good reliability. In addition, for both parts, the entire survey had a reliability rate of more than 0.7, indicating that both parts had good reliability and accuracy (Table 4).

**Table 4:** The results of internal consistency in both parts of the survey

Survey	Factor	N (question)	Cronbach’s Alpha	McDonald’s Omega coefficient
Part A	Necessary care	13	0.890	0.900
	Supportive care	5	0.745	0.780
	Secondary care	6	0.794	0.800
	**Part A**	**24**	**0.933**	**0.940**
Part B	Labor resource	3	0.726	0.740
	Material resource	3	0.837	0.850
	Communication	4	0.884	0.890
	Responsibility	4	0.829	0.830
	Unpredictable situations	3	0.734	0.730
	**Part B**	**17**	**0.910**	**0.910**

## Discussion

In this study, we translated the MISSCARE Survey and then assessed its internal reliability and construct validity to create a valid Persian version of the survey.

When we began our assessment of content validity, some of the experts in our study believed that some items in Part A were not useful in Iran. However, we retained those items, and after assessing nurses’ responses to them as well as the construct validity, we decided to keep the items in the survey. Thus, no items from either part of the survey were removed. In the translation of the MISSCARE Survey- Swedish version, the authors encountered numerous difficulties with certain items because 15 years had passed since the creation of the original survey; they thus removed six items from Part B [10]. Finding a term that describes “MISSCARE” was difficult in the Icelandic language, and the researchers working on the Icelandic version also had trouble explaining how participants should answer the items [13]. We encountered this problem as well; in some cases, we received a returned questionnaire and found that the participants answered the items in a manner that was the opposite of what was expected.

We divided both parts of the survey into different factors for construct validity. The Persian version of the MISSCARE Survey has three factors in Part A and five factors in Part B. Studies have been conducted in the US, Turkey, and Part A does not have any factors; rather, it is simply a list of nursing care activities [2, 10–13]. Zelenikova et al. divided Part A into four factors (assessment, individual needs, basic care, and planning) that could better assess the extent of missed nursing care [9]. Most of the studies divided Part B into three factors: labor resources, material resources, and communication. We divided Part B into five factors (labor resources, material resources, communication, responsibility, and unpredictable situations) to more accurately determine the reasons for missed care. In addition, the MISSCARE- BRASIL has five factors for Part B (labor resources, material resources, communication, ethics, and management) [12].

Both parts of the MISSCARE Survey- Persian version has good internal consistency, and Cronbach’s alpha was 0.933 for Part A and 0.910 for Part B. The minimum required was 0.7. Cronbach’s alpha for Part A in our study was higher than that in some former studies, such as the United States, Slovakia, and Iceland [2, 9, 13], but it was smaller than that obtained in Turkey’s study [11]. For Part B, it was higher than that in studies such as those in the United States, Turkey, Sweden, and Iceland [2, 10, 11, 13] but smaller than that reported in the Slovakia study [9].

In Iran, the lowest degree in nursing is a bachelor’s degree. Thus, most of our participants had bachelor’s degrees, and some had master’s degrees in nursing. In studies conducted in the United States, Slovakia, and Brazil, most participants were technicians and had an education level below a bachelor’s degree [2, 9, 12].

Based on our results, each nurse cares for 1–4 patients in closed units (ICU and CCU) and 6–12 patients in other units. Kalisch et al. and Siqueira et al. stated that in their studies, each nurse cared for 1–2 patients in closed units and 5–8 to patients in the surgical unit [2, 12]. There is a nursing shortage in Iran, and nurses there have to care for several patients during each shift. Missed nursing care may increase in a critical situation such as the COVID-19 pandemic; our results regarding the COVID-19 unit showed that supportive care was missed more often in that unit than in other units.

Additionally, the perception of staffing adequacy is positively associated with job satisfaction [17]. In our study, nurses’ satisfaction was assessed in three domains (current position, being a nurse, and teamwork). Our results showed that only 14.6% of nurses were very satisfied or satisfied in their current position, while 25.3% were very satisfied or satisfied with being a nurse, and 30.7% were very satisfied or satisfied with the teamwork in their unit. The level of satisfaction in all three domains was reported to be high in Siqueira et al.’s study, in contrast to the missed-care results in this study [12]. In the Slovakia study, researchers reported a low level of satisfaction in all domains [9]. In all studies, the level of satisfaction with teamwork was higher than that concerning the other domains. Some studies have shown that nurses feel more dissatisfied with their jobs when they cannot take care of their patients. In other words, the more missed nursing care in the unit, the higher the nurses’ dissatisfaction level about their job [17–19].

Emotional support for the patient and/or family, feeding patients when the food is still warm, and attending interdisciplinary care conferences whenever held were missed more often than other types of care in our study. In addition, missed handwashing, completion of documentation of all necessary data, and monitoring of intake/output were reported as minor. Zelenikova et al. reported that feeding patients when the food is still warm and attending interdisciplinary care conferences whenever held were missed more often in their study [9]. However, Kalisch stated that ambulation three times per day or as ordered, turning patients every 2 h, and assessing the effectiveness of medications were missed more often in their study [2]. Handwashing was one of the least missed types of care in the United States and Turkey studies [2, 11].

The findings of our study showed that the reasons for missed nursing care were inefficient communication, labor resources, responsibility, unpredictable situations, and material resources, respectively. In some studies, such as those in the United States, Turkey, and Sweden, inefficient communication was the most fundamental reason for missed nursing care [2, 10, 11], but studies in Slovakia and Brazil reported that labor resources were the most critical reason in those countries [9, 12].

### Limitations

This study was conducted in only one province, and the results may not be generalizable to all provinces due to the cultural diversity that exists among Iran’s different provinces. The participants were chosen from five units, and the results may differ in some units, such as the emergency department. Further study could focus on the development or psychometrics of a tool that assesses missed care from the patient’s perspective. Such a tool may be more helpful in identifying problems and their solutions.

## Conclusion

The MISSCARE Survey- Persian version has good validity, reliability, and psychometric properties. It can help assess missed care in hospitals as well as the reasons care is missed. The MISSCARE Survey- Persian version also has different domains in the two parts of the survey, which can be helpful in better identifying the missed care and the exact reasons it happens. On the other hand, nursing managers might use this questionnaire to solve many problems related to caring and organization. This may result in provision of the best quality of care and satisfied nursing staff.

## Data Availability

The datasets used and analyzed during the current study are available from the author based on reasonable request.

## Abbreviations

Missed care: Care that is reduced, delayed, or eliminated
CVR: Content Validity Ratio
CVI: Content Validity Index
RMSEA: Root Mean Square Error of Approximation
CFA: Confirmatory factor analysis
GFI: Goodness of fit index
NFI: Normed Fit Index
CFI: Comparative Fit Index
